# Natural Killer Cells Generated from Cord Blood Hematopoietic Progenitor Cells Efficiently Target Bone Marrow-Residing Human Leukemia Cells in NOD/SCID/IL2Rg^null^ Mice

**DOI:** 10.1371/journal.pone.0064384

**Published:** 2013-06-05

**Authors:** Jeannette Cany, Anniek B. van der Waart, Marleen Tordoir, Gerben M. Franssen, Basav N. Hangalapura, Jolanda de Vries, Otto Boerman, Nicolaas Schaap, Robbert van der Voort, Jan Spanholtz, Harry Dolstra

**Affiliations:** 1 Department of Laboratory Medicine, Laboratory of Hematology, Radboud University Nijmegen Medical Centre (RUNMC), Nijmegen, The Netherlands; 2 Glycostem Therapeutics, Nijmegen, The Netherlands; 3 Department of Nuclear Medicine, RUNMC, Nijmegen, The Netherlands; 4 Department of Tumor Immunology, Nijmegen Centre for Molecular Life Sciences, Nijmegen, The Netherlands; 5 Department of Hematology, RUNMC, Nijmegen, The Netherlands; Centre de Recherche Public de la Santé (CRP-Santé), Luxembourg

## Abstract

Natural killer (NK) cell-based adoptive immunotherapy is an attractive adjuvant treatment option for patients with acute myeloid leukemia. Recently, we reported a clinical-grade, cytokine-based culture method for the generation of NK cells from umbilical cord blood (UCB) CD34^+^ hematopoietic progenitor cells with high yield, purity and *in vitro* functionality. The present study was designed to evaluate the *in vivo* anti-leukemic potential of UCB-NK cells generated with our GMP-compliant culture system in terms of biodistribution, survival and cytolytic activity following adoptive transfer in immunodeficient NOD/SCID/IL2Rg^null^ mice. Using single photon emission computed tomography, we first demonstrated active migration of UCB-NK cells to bone marrow, spleen and liver within 24 h after infusion. Analysis of the chemokine receptor expression profile of UCB-NK cells matched *in vivo* findings. Particularly, a firm proportion of UCB-NK cells functionally expressed CXCR4, what could trigger BM homing in response to its ligand CXCL12. In addition, high expression of CXCR3 and CCR6 supported the capacity of UCB-NK cells to migrate to inflamed tissues via the CXCR3/CXCL10-11 and CCR6/CCL20 axis. Thereafter, we showed that low dose IL-15 mediates efficient survival, expansion and maturation of UCB-NK cells *in vivo*. Most importantly, we demonstrate that a single UCB-NK cell infusion combined with supportive IL-15 administration efficiently inhibited growth of human leukemia cells implanted in the femur of mice, resulting in significant prolongation of mice survival. These preclinical studies strongly support the therapeutic potential of *ex vivo*-generated UCB-NK cells in the treatment of myeloid leukemia after immunosuppressive chemotherapy.

## Introduction

Acute myeloid leukemia (AML) is a clonal disorder characterized by the accumulation of abnormal myeloid progenitor cells and suppression of normal hematopoiesis [1]. With a median age of ∼70 years at diagnosis [2], AML is most common in the elderly and its yearly incidence is expected to increase as the population ages [3]. Current chemotherapeutic regimens lead to remission rates of 60–85%. However, relapse occurs in the vast majority of AML cases, resulting in a 5-year overall survival of 40% in patients <60 years of age, which even drops to 10% in elderly patients due the higher prevalence of bad risk cytogenetics and poor chemotherapeutic tolerance [4]. Although allogeneic stem cell transplantation (alloSCT) is potentially curative, mostly younger patients can benefit from this therapeutic option due to high association with transplant-related morbidity and mortality [5]. Therefore, adjuvant and alternative treatment options are urgently needed.

Transfusion of allogeneic NK cells is a promising therapeutic approach for patients with AML. NK cells are major effector cells of the innate immune system and play a key role in control against virus infection and tumor immunosurveillance [6], [7]. In the setting of haploidentical alloSCT, NK cell alloreactivity has proven to decrease relapse rate and improve survival among AML patients [8]. Therefore, there is an emerging interest in exploiting adoptive NK cell transfer in the treatment of AML. Clinical studies reported so far showed that infusion of haploidentical NK cells derived from leukapheresis products resulted in objective clinical responses in high-risk AML patients [9], [10], as well as long term remissions in childhood AML [11].

However, further improvement of NK cell-based therapy is needed to increase the clinical effect. In this regard, NK cells generated *ex vivo* from hematopoietic progenitor cells (HPC) may have significant clinical benefits over enriched NK cells from adult donors, including the ability to choose an appropriate killer-cell immunoglobulin-like receptor (KIR)-ligand or KIR B haplotype alloreactive donor, as well as the capacity to reach high therapeutic dosages. Recently, we reported a GMP-compliant, cytokine/heparin-based culture protocol for the *ex vivo* generation of highly active NK cells from CD34^+^ HPC isolated from cryopreserved umbilical cord blood (UCB) units [12]. Expansion in closed, large-scale bioreactors yields a clinically relevant dose of NK cells with high purity and cytolytic activity against AML cells *in vitro* [13].

In the present study, we aimed at evaluating the anti-leukemic potential of UCB-NK cells *in vivo* in terms of biodistribution, survival and cytotoxicity following adoptive transfer in NOD/SCID/IL2Rg^null^ (NSG) mice. Therefore, we established an ^111^Indium labelling protocol that enables specific and sensitive *in vivo* tracking of infused UCB-NK cells by single photon emission computed tomography (SPECT) imaging. Besides generating insight in UCB-NK cell trafficking, we demonstrated specific accumulation of UCB-NK cells in the bone marrow (BM) that matched their chemokine receptor profile. Moreover, we demonstrated that a single infusion of UCB-NK cells resulted in potent leukemia cell growth inhibition and significantly improved mice survival. These findings strongly support *ex vivo*-generated UCB-NK cells as promising immunotherapeutic products for the treatment of AML.

## Materials and Methods

### UCB-NK Cell Generation

UCB units were obtained at birth after normal full-term delivery after written informed consent with regard of scientific use from the cord blood bank of the Radboud University Nijmegen Medical Centre (RUNMC, Nijmegen, The Netherlands). The use of UCB units was approved by the RUNMC Institutional Review board. NK cells were generated from cryopreserved UCB-derived HPC as previously reported [12], [13]. Briefly, expanded CD34^+^ UCB cells were differentiated and further expanded using NK cell differentiation medium which consists of Glycostem Basal Growth Medium (GBGM®) for cord blood (Clear Cell Technologies) supplemented with 2% human serum (HS; Sanquin Blood Supply Foundation, Nijmegen, The Netherlands), low-dose GM-CSF (Neupogen), G-CSF, IL-6 (both CellGenix) and a high-dose cytokine cocktail consisting of IL-7, SCF, IL-15 (all CellGenix) and IL-2 (Proleukin®). The cell density was checked two times a week and adjusted to >1×10^6^ cells/ml by the addition of GBGM® NK cell differentiation medium. For experiments, CD56^+^CD3^−^ UCB-NK cells were used at the end of the culture process with >90% purity, what was typically achieved within 3–4 weeks in GBGM® NK cell differentiation medium.

### Flow Cytometry

Cell numbers and expression of cell surface markers were determined by flow cytometry. Anti-human CD45-ECD (J.33) and CD56-PC7 (N901) antibodies (Beckman Coulter) were used to follow cell number and NK cell differentiation during culture using the Coulter FC500 flow cytometer (Beckman Coulter). The population of living CD45^+^ cells was determined by exclusion of 7AAD (Sigma) positive cells. For phenotypical analysis, UCB-NK cells were incubated with the appropriate concentration of antibodies for 30 min at 4°C. After washing, cells were resuspended in PBS/0.5% BSA and analyzed using the Coulter FC500 or Cyan-ADP 9 color flow cytometers (Beckman Coulter). The following conjugated monoclonal antibodies were used: CCR2 (48607, R&D system), CCR5 (T21/8, eBioscience), CCR6 (TG7), CCR7 (G043H7), CXCR3 (G025H7), CXCR4 (12G5), CXCR6 (TG3), CX3CR1 (2A9–1) and CD62L (DREG-56, all Biolegend).

### *In vitro* Cell Migration Assay

UCB-NK cells were resuspended in GBGM/2% HS and loaded into transwell inserts (10^5^ cells/well, 5 µm pore filter transwell, 24-well plate, Corning). The human chemokines CCL4, CCL20, CXCL10, CXCL11 and CXCL12 (all Immunotools) were diluted at 10–250 ng/ml and added to the lower compartment (600 µl/well) in triplicates. After 2 h at 37°C, inserts were removed; cells in lower compartments were collected, stained for CD56 and quantified by flow cytometry. Percentage of migrated cells was calculated as the number of CD56^+^ cells in the lower compartment divided by the total number of CD56^+^ loaded cells.

### Mice

NOD/SCID/IL2Rg^null^ (NSG) mice were originally purchased from Jackson Laboratories, and housed and bred in the RUNMC Central Animal Laboratory. Male NSG mice were used from 6 to 12 weeks of age (weight was 20–30 g). All animal experiments were approved by the Animal Experimental Committee of the RUNMC and were conducted in accordance with institutional and national guidelines under the university permit number 10300.

### NK Cell Labeling with ^111^Indium, SPECT-CT Imaging and Biodistribution Analysis

UCB-NK cells were labeled with ^111^Indium-oxinate (^111^In; GE Healthcare) in PBS Tris 0.1 M HCl, pH 7.4 for 15 min at RT at doses mentioned in the text. After incubation, cells were washed twice with PBS/2% HS and resuspended in PBS before use. Viability was assessed by trypan blue exclusion and cell-associated activity was quantified using a dose calibrator VDC-404 (Veenstra Instruments, The Netherlands). Lysates were obtained after three freezing/thawing cycles of ^111^In-NK cells previously resuspended in distilled water. Whole body scans of isoflurane gas anesthetized (2% in air) mice were acquired with a SPECT-CT dual-modality scanner (U-SPECT II, MiLabs) for 30–45 min using a 1.0 mm diameter pinhole mouse collimator cylinder. Scans were reconstructed with MiLabs reconstruction software and analyzed using Inveon Research Workplace software. For biodistribution analysis, mice were euthanized by cervical dislocation, tissues of interest were dissected, weighed, and analyzed for their ^111^In content using a shielded 3-inch-well-type gamma counter (Wizard; Pharmacia LKB). The ^111^In activity in each tissue was expressed as percentage of the injected dose (%ID) per gram of tissue and was normalized to the blood level. Values for the total blood and BM fraction were extrapolated according to physiological values, with blood being 6% of the total body weight, and one femur being 6.7% of the total BM fraction [14].

### Intra-femoral K562 Model, Bioluminescence Imaging and UCB-NK Cell Adoptive Transfer

The NK-sensitive leukemia cell line K562 (ATCC) was cultured in Iscove's modified Dulbecco's medium (IMDM; Invitrogen) containing 50 U/ml penicillin, 50 µg/ml streptomycin and 10% fetal calf serum. Green fluorescent protein (GFP) and Luciferase expressing K562 cells (K562.LucGFP) were generated by stable transduction of parental cells with lentiviral particles LVP20 encoding the reporter genes under control of the CMV promoter (GenTech). To establish a preclinical AML xenograft model, adult NSG mice were injected in their right femur with 10^5^ K562.GFPLuc cells (injection volume = 5 µl), by insertion of a 25G Hamilton needle through the knee joint of isoflurane gas anesthetized mice. Using this procedure, leukemia cell growth remained localized to BM up to 5 weeks. Thereafter, tumor cells eventually overgrow outside the bone forming a palpable tumor. Mice were sacrificed (cervical dislocation) when the palpable tumor reached 1 cm in diameter or when one of the following criteria was observed: severe weight loss, poor coat and skin condition, static activity or paraplegia. Leukemia load was monitored by bioluminescence imaging (BLI) following injection of Luciferine (3.5 mg per mouse, Caliper Life Science) using the IVIS system (Xenogen). Images were analyzed using Living Image Software 2.5 (Xenogen). Leukemia load was quantified in the region of interest with subtraction of background signal, and expressed as photons per second. For adoptive transfer, UCB-NK cells were resuspended in PBS and injected *i.v.* via the tail vein of adult NSG mice. Recombinant human IL-15 (Miltenyi Biotech) was administrated intra-peritoneally (*i.p.*) at the dose of 0.5 µg/mouse (*i.e.* 2.500 Units/mouse) the day of UCB-NK cell infusion and thereafter every 2–3 days for 2 weeks.

### Statistical Analysis

Statistical analyses were performed using Graphpad Prism 5 software. Biodistribution of ^111^In-associated activity following ^111^In-NK cell infusion was compared to that observed after lysate or free ^111^In injection using one way-analysis of variance (ANOVA) and Dunnett’s multiple comparison tests. Two way-ANOVA followed by Bonferroni post-hoc test, and log rank Mantel Cox tests were used in anti-leukemic studies as indicated in the figure legends. Differences were considered to be significant for *p* values <0.05.

## Results

### Characterization of the Homing Receptor Expression Profile of UCB-NK Cells

**Figure 1:**
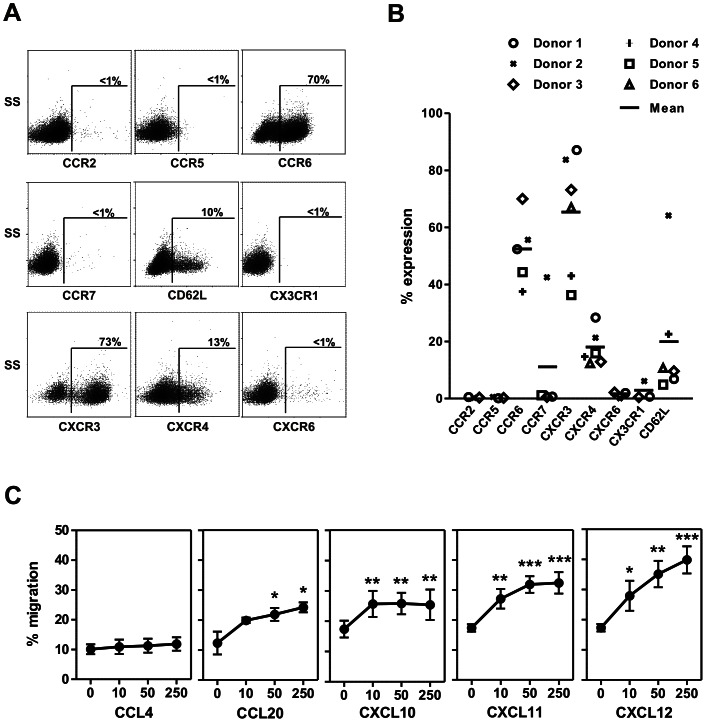
Homing receptor expression profile of UCB-NK cells. (A–B) The expression level of homing receptors was analysed by flow cytometry on UCB-NK cells at the end of the culture process. (A) Dot plots gated on CD56^+^ cells from one representative donor; (B) Summary of 6 different donors analysed. (C) The capacity of UCB-NK cells to respond *in vitro* to gradients (10 to 250 ng/ml) of the chemokines CCL4 (CCR5 ligand), CCL20 (CCR6 ligand), CXCL10, CXCL11 (both CXCR3 ligand) and CXCL12 (CXCR4 ligand) was evaluated in transwell migration assays as described in materials and methods. Mean ± SEM of three independent experiments are shown, each performed with different UCB-NK cell donors. Migration towards specific chemokines was compared to non-specific migration (0 ng/ml) using a one way-ANOVA followed by Dunnett’s multiple comparison post-hoc test, *p<0.05, **p<0.01, ***p<0.001.

We reported previously that the cytokine-based culture system that we established allows generation of CD3^−^CD56^+^ NK cells with high purity, that express typical inhibitory and activating NK receptors and display similar cytotoxic gene expression profile to peripheral blood NK cells [15]. To further characterize UCB-NK cell products and to investigate their biodistribution potential upon adoptive transfer, we examined the expression and functionality of a panel of receptors that were previously described to participate in the regulation of human NK cell trafficking *in vivo* [16], [17]. As shown in Figure 1A–B, the chemokine receptors CCR2, CCR5, CCR7, CXCR6 and CX3CR1 were virtually absent. However, high proportion of UCB-NK cells expressed CCR6 (52±12%) and CXCR3 (65±21%). Expression of CXCR4 as well as the adhesion molecule CD62L (L-selectin) were also detected typically on 10–20% of UCB-NK cells at the end of the culture process. Results obtained from *in vitro* migration assays were consistent with this chemokine receptor profile (Figure 1C). Notably, the proportion of CD56^+^ UCB-NK cells migrating in response to the chemokine CXCL12 was similar to level of CXCR4-expressing cells, and specific migration towards the chemokines CCL20 (CCR6 ligand), CXCL10 and CXCL11 (both CXCR3 ligands) were confirmed. These data indicate that UCB-NK cells functionally express CXCR4, what could trigger BM homing in response to its ligand CXCL12. In addition, data suggest that UCB-NK cells have the capacity to migrate to inflamed tissues *via* the CXCR3/CXCL10–11 and CCR6/CCL20 axis.

### Development of *in vivo* UCB-NK Cell Tracking

**Figure 2:**
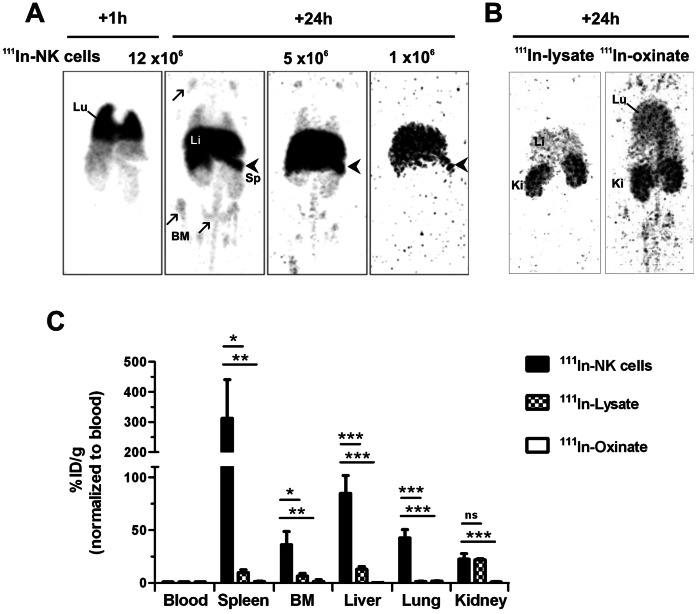
SPECT-CT imaging provides good sensitivity and specificity to track ^111^In-labeled UCB-NK cells ***in vivo*** **.** To address the feasibility of UCB-NK cell tracking *in vivo*, adult NSG mice were injected *i.v.* either with increasing doses of ^111^In-NK cells (1, 5 or 12×10^6^ ^111^In-NK cells per mouse equivalent to 1, 5 and 12 MBq respectively, n = 5 in total), with a lysate obtained from ^111^In-NK cells (^111^In-lysate, n = 2) or with ^111^In-oxinate (n = 3). (A) Representative whole body SPECT scans acquired 1 h and 24 h after injection of ^111^In-NK cells with major visualization of lungs (Lu), liver (Li), spleen (Sp, arrowhead) and bone marrow (BM, arrow). (B) Representative whole body SPECT scans acquired 24 h after injection of ^111^In-NK cell lysate or ^111^In-oxinate; kidneys (Ki), heart (He). (C) Quantitative biodistribution analysis performed 24 h after injection. For comparison, the relative ^111^In-content of each tissue of interest was normalized to blood. Data are shown as mean ± SD. *p<0.05, **p<0.01, ***p<0.001.

Next, we aimed to establish a method using ^111^In-oxinate as radiolabel and SPECT-CT imaging that could be exploited both at the pre-clinical level in mice and for clinical studies in humans, to monitor early distribution of UCB-NK cell following infusion in relation to anti-leukemic potency in BM. To address the feasibility of this methodology, increasing doses of UCB-NK cells previously labeled with ^111^In-oxinate (^111^In-NK cells; 2MBq of ^111^In-oxinate was added per 10^6^ cells and labeling efficiency was 55%) were injected *i.v.* into NSG mice. Whole body scans acquired 1 and 24 h after infusion showed that the ^111^In-activity first localized in the lungs, and thereafter redistributed to the spleen, liver and BM (Figure 2A). At 24 h, liver and spleen were visible at all doses of infused ^111^In-NK cells, while the activity present in BM was detected only in mice injected with ≥5×10^6^ ^111^In-NK cells. To address the specificity of ^111^In-NK cell *in vivo* imaging, we also analyzed the distribution of ^111^In-activity following injection of either a lysate obtained from ^111^In-NK cells or ^111^In-oxinate (Figure 2B). In both cases, after 24 h, the activity was mainly visualized in kidneys. Weak uptake of ^111^In was noticed in liver and lungs following injection of ^111^In-lysate and ^111^In-oxinate, respectively.

**Figure 3:**
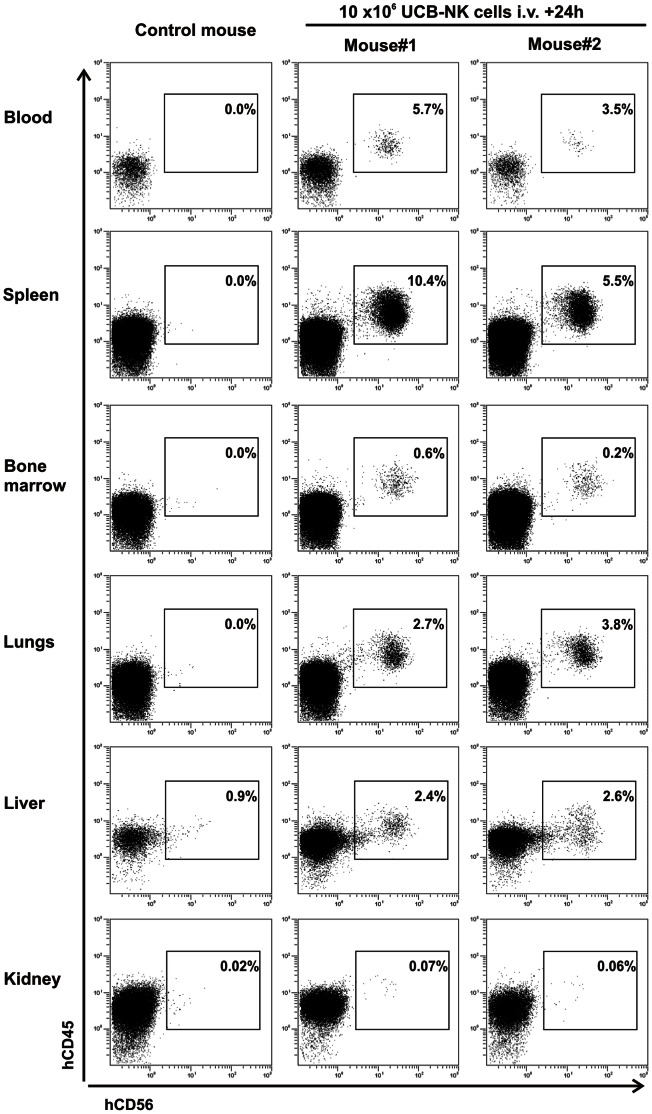
*Ex vivo* flow cytometric analysis confirmed trafficking of UCB-NK cells through lymphoid tissues, liver and lungs following adoptive transfer in NSG mice. Two adult NSG mice were infused *i.v.* with 10×10^6^ UCB-NK cells. The day after, mice were sacrificed, organs collected and used to prepare cell suspension for *ex vivo* flow cytometric analysis following erythrocyte lysis. One additional non-injected mouse was used as control. Presence of human CD45^+^CD56^+^ NK cells was confirmed in all examined tissues except kidneys. Dot plots gated on total living cells are shown.

After imaging, mice were euthanized, organs were collected, weighted, and used to analyse quantitatively the distribution of the ^111^In-activity as described in materials and methods. All tissues examined following injection of ^111^In-oxinate exhibited similar level of ^111^In compared to that measured in blood, indicating that the ^111^In activity remained in the circulation, resulting in visualization of well perfused organs. In mice injected with ^111^In-lysate, the activity mainly accumulated in the kidneys, representing ∼20% of the injected dose (data not shown). Slightly enhanced ^111^In activity levels were also measured in liver and lymphoid organs. In contrast, following infusion of ^111^In-NK cells, the proportions of activity quantified in BM, spleen, liver and lungs were strongly increased compared to blood level. Values were also significantly higher compared to those determined following injection of ^111^In-lysate and ^111^In-oxinate (Figure 2C), indicating that the accumulation of ^111^In activity in these organs could be attributed to the accumulation of ^111^In-NK cells. Notably, the distribution of ^111^In activity was similar at all doses of infused ^111^In-NK cells (Figure S1). Moreover, human CD45^+^CD56^+^ NK cells were clearly identified in the same organs, except kidneys, by *ex vivo* flow cytometric analysis performed 24 h after infusion of UCB-NK cells (Figure 3). All together, these data show that SPECT-CT imaging allows tracking of ^111^In-NK cells *in vivo* with good sensitivity and specificity, and that UCB-NK cells, after a brief period of retention in the lungs, rapidly traffic to the liver, spleen and BM.

### A Consistent Proportion of UCB-NK Cells Accumulates in Mouse BM Following Adoptive Transfer

**Figure 4:**
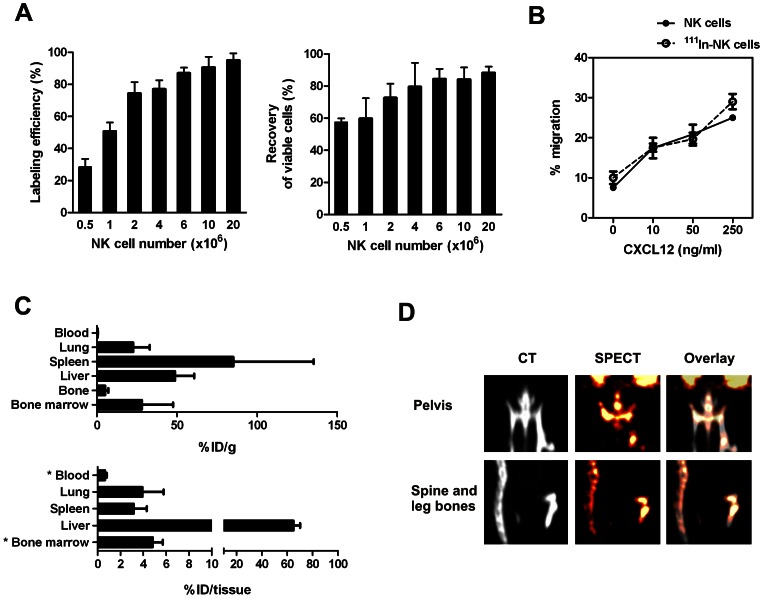
Organ-distribution of UCB-NK cells following adoptive transfer. (A) For optimization of NK cell labelling procedure, increasing number of UCB-NK cells were incubated with 2MBq ^111^In. Graphs show mean ± SD of three experiments performed each with a different UCB donor. Good labelling efficiency and cell recovery were achieved using >4×10^6^ cells per 2 MBq. Subsequently (panels B-D), 0.4MBq ^111^In was added per 10^6^ UCB-NK cells to label. (B) The capacity of ^111^In-NK cells to migrate in response to the chemokine CXCL12 was evaluated *in vitro* and compared to that of unlabeled UCB-NK cells. The same experimental procedure was employed to assess the transwell migration of unlabeled and ^111^In-NK cells as described in materials and methods, except that the proportion of ^111^In-labeled cells present in bottom chambers were quantified using a shielded 3-inch-well-type gamma counter (Wizard; Pharmacia LKB). (C) Biodistribution analysis of ^111^In-NK cells 24 h after *i.v.* infusion, expressed as a percentage of injected activity per gram of tissue of interest or per organ. Combined results from two experiments expressed as mean ± SD (n = 6) are shown. *Extrapolated values according to physiological parameters. (D) Representative 2D-reconstruction analysis of SPECT-CT illustrating the accumulation of ^111^In-NK cells (orange) in bones (grey).

After demonstrating the feasibility of ^111^In-NK cell tracking *in vivo*, we aimed to determine quantitatively the biodistribution of UCB-NK cells in 2 independent experiments. To this end, we first optimized the labelling procedure by incubating increasing numbers of NK cells with 2 MBq ^111^In. Good ^111^In labelling efficiency and cell recovery were achieved using >4×10^6^ cells (Figure 4A). Based on these findings, 0.4 MBq of ^111^In-oxinate was added per 10^6^ cells for subsequent experiments where labeling efficiency always exceeded 80%, cell viability >90% and cell recovery >95% (data not shown). In addition, this procedure did not affect the migration capacity of UCB-NK cells towards the prototypic BM-chemokine CXCL12 *in vitro* (Figure 4B).

As observed in the pilot studies, we found the highest activity concentration in the spleen, followed by liver, BM and lungs at 24 h after infusion. Taking into account total organ weights, nearly 70% (64.9±2.1) of the ID was present in liver, while spleen and lungs contained 3.1±1.2%ID and 3.9±1.9%ID, respectively (Figure 4C). For BM, 0.32±0.05%ID was quantified per femur (data not shown). Assuming that one femur accounts for 6.7% of the total BM in adult mouse [14], we estimated at ∼5% the fraction of ^111^In-NK cells accumulating within 24 h in the total BM compartment. Accordingly, a homogenous ^111^In-signal was visualized by SPECT-CT imaging in all bones (Figure 4D). These data indicate that a significant percentage of UCB-NK cells are able to migrate to the mouse BM following adoptive transfer.

### Low Dose Human IL-15 Drives UCB-NK Cell Expansion *in vivo*

**Figure 5:**
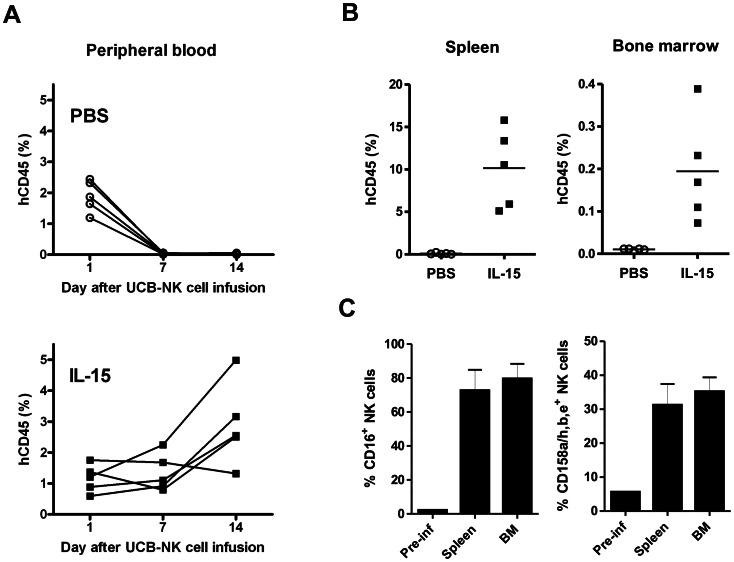
Low-dose IL-15 mediates efficient UCB-NK cell survival and expansion *in vivo*. Adult NSG mice were injected *i.v.* with 5×10^6^ UCB-NK cells with or without supportive IL-15. Recombinant human IL-15 was administered every 2–3 days for 2 weeks at the dose of 0.5 µg/mouse/injection, starting the day of UCB-NK cell infusion. (A) Percentages of circulating human CD45^+^ cells in all CD45^+^ cells were quantified by flow cytometry at the indicated time points. Each line corresponds to one mouse. (B) Percentages of human CD45^+^ cells in all CD45^+^ cells quantified in spleen and bone marrow (leg bones) 2 weeks after UCB-NK cell infusion. (C) Expression of CD16 and CD158a/h,b,e on UCB-NK cells before and 2 weeks after infusion into NSG mice. Percentages were determined on human CD45^+^CD56^+^ NK cells isolated from spleen and bone marrow. Graphs show the mean ± SD of 5 mice.

Next to BM homing, we aimed to evaluate the survival potential of UCB-NK cells following adoptive transfer. Indeed, clinical responses reported so far always occurred with concomitant donor NK cell persistence and even expansion within the first two weeks after infusion. Interestingly, conditioning regimens that allowed successful alloNK cell engraftment also resulted in transient elevation of endogenous IL-15 [9]. Therefore, we examined UCB-NK cell survival potential upon adoptive transfer in NSG mice in the presence of low dose IL-15 support. In a first experiment, we observed that daily administration of IL-15 mediated efficient expansion of infused UCB-NK cells *in vivo* (Figure S2). In contrast to mice injected with UCB-NK cells alone, a clear population of human CD45^+^CD56^+^ cells could be visualized in mice co-injected with IL-15, representing 3.5% of total leukocytes at day 7, which further increased till 4.5% at day 14. Nevertheless, NK cell numbers rapidly declined after removal of IL-15 (Figure S2). We next intended to confirm these findings with a different UCB donor and to examine UCB-NK cell engraftment level in lymphoid tissues. To this end, mice received a single infusion of 5×10^6^ UCB-NK cells, and percentages of circulating human NK cells were monitored in peripheral blood collected at 1, 7 and 14 days later. Here, NSG mice were given intermittent injections of IL-15, thereby reducing the IL-15 support compared to the first experiment. Consistent with previous observations, human NK cells were almost absent at day 7 in mice co-treated with PBS. In contrast, mice co-injected with IL-15 displayed stable levels of NK cells from day 1 to day 7, which even increased in 4 out 5 mice at day 14 (Figure 5A). In addition, significant percentages of UCB-NK cells were identified in spleen and BM by day 14 (Figure 5B). Moreover, examination of the NK cell phenotype in these tissues revealed high expression level of CD16 and KIR receptors, which were strongly increased compared to the infused UCB-NK cell product (Figure 5C). All together, these data demonstrate that low dose IL-15 mediates efficient UCB-NK cell survival and expansion *in vivo*, and that further differentiation of UCB-NK cell products occurs rapidly following adoptive transfer.

### Adoptive Transfer of UCB-NK Cells Inhibits Growth of BM-residing Human Leukemia Cells in Mice

**Figure 6:**
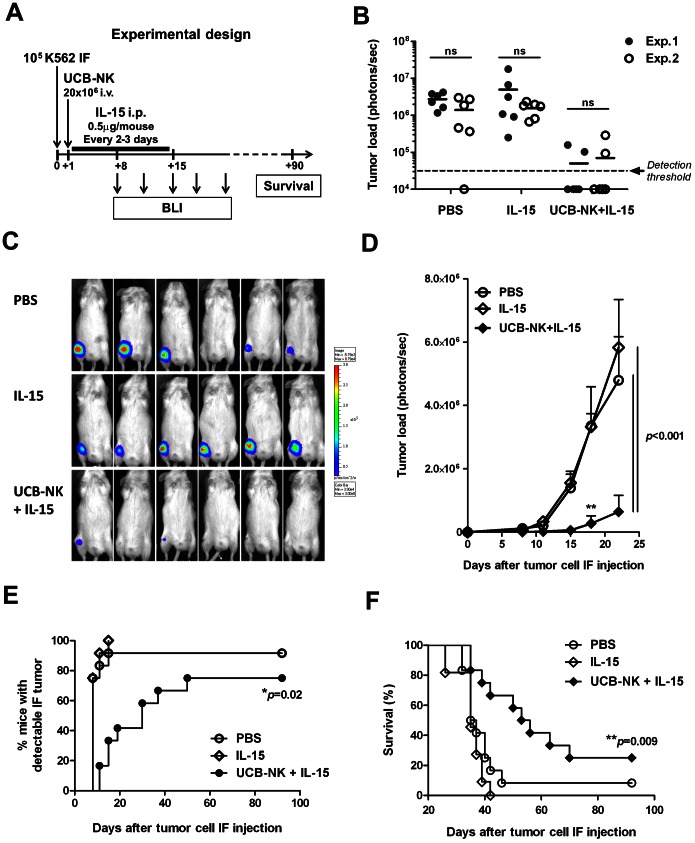
A single infusion of UCB-NK cells inhibits growth of BM-residing human leukemia cells. The potential of UCB-NK cells to attack human leukemia *in vivo* was evaluated in NSG mice bearing K562 intra-femoral (*i.f*.) tumors. (A) Experimental study design: adult NSG mice were injected in their right femur with 10^5^ K562.LucGFP cells. The day after, mice were treated with 20×10^6^ UCB-NK cells *i.v.* in combination with IL-15 administration (0.5 µg/mouse *i.p.* every 2–3 days for 14 days), or received PBS or IL-15 alone as control (n = 6 per group). Tumor load was monitored by BLI from day 8 after K562.LucGFP cell inoculation and next every 3–4 days for 2 weeks. At later time points, only mice with undetectable tumor load were imaged. (B) Two independent anti-leukemic studies were performed with similar outcomes. Tumor load per mouse measured at day 15 following K562.LucGFP cell IF injection from experiment 1 (black circles) and 2 (open circles). (C) BLI pictures acquired at day 15 after tumor cell *i.f.* injection, and (D) tumor load in time (mean ± SD, n = 6 per group). **p<0.01 UCB-NK cells+IL-15 vs. PBS and IL-15. Data of experiment 2 are shown. (E) Time to first tumor detection by BLI and (F) mice survival analysed according to Mantel Cox test. One mouse from the IL-15 group (experiment 1) died at day 19 after luciferine injection and was excluded from the survival analysis.

Finally, to evaluate the cytolytic activity of UCB-NK cells *in vivo*, we developed a leukemia xenograft model by injecting adult NSG mice with leukemia cells intra-femorally (*i.f.*), therefore requiring BM homing by infused UCB-NK cells to achieve anti-leukemic response. For this, we used K562 leukemia cells expressing Luciferase (K562.LucGFP cells) to allow longitudinal monitoring of tumor growth in living animals by bioluminescence imaging (BLI). In this model, mice received a single infusion of UCB-NK cells the day after K562.LucGFP cell *i.f.* injection, in combination with low dose supportive IL-15 (Figure 6A). A dose of 20×10^6^ NK cells per mouse was defined based on biodistribution studies to approach an effector to target ratio of 1∶1 *in vivo*. The anti-leukemic potential of UCB-NK cells to target K562.LucGFP cells was evaluated in two independent experiments with similar outcomes (Figure 6B). Comparable K562 cell engraftment was observed in both experiments and all control mice treated with PBS and IL-15 except one displayed detectable *i.f.* tumors by day 15 (Figure 6B–C). In striking contrast, 8 out of 12 mice treated with UCB-NK cells had undetectable tumor load by day 15. Significant inhibition of K562.LucGFP cell progression following UCB-NK cells infusion was demonstrated by BLI in time (Figure 6D). Most importantly, a single infusion of *ex vivo*-generated UCB-NK cells in combination with low-dose IL-15 prolonged survival of K562 *i.f.* injected mice of which 25% showed long-term protection (Figure 6E–F). These results demonstrate that UCB-NK cells are functional following adoptive transfer, and that they are able to target and eliminate BM-residing leukemia cells *in vivo.*

## Discussion

To date, it is well established that NK cells mediate efficient graft-versus-leukemia reactivity with improved control of relapse in AML patients following alloSCT. This raises the interest in exploiting NK cells for adoptive immunotherapy, particularly as an adjuvant treatment approach to chemotherapy for elderly, high-risk and refractory AML patients. Most trials reported so far employed peripheral blood derived-NK cells enriched by CD3 depletion with or without CD56 selection from donor apheresis products, and showed that enriched NK cell infusions are well tolerated, without induction of GVHD or severe toxicity [9]–[11], [18]. Nevertheless, the clinical impact of NK cell-based therapy remains inconsistent and several issues need to be optimized to achieve clinical efficacy. In addition to conditioning regimens that prevent rapid graft rejection and supply of exogenous cytokines like IL-2 supporting NK cell survival upon adoptive transfer, the purity, the activation and the number of cells that can be infused in patients are critical factors determining clinical response [9], [11]. Since, new methodologies are currently emerging to generate higher numbers of activated NK cells, including *in vitro* expansion of donor-derived NK cells before infusion [19], as well as NK cell generation from CD34^+^ hematopoietic stem/progenitor cells [12], [20], [21]. In particular, the availability of cryo-preserved UCB units and the development of GMP-compliant culture systems constitute a very attractive approach to exploit the potential of NK cell-based adoptive immunotherapy, with generation of a clinically relevant dosages of UCB-NK cells with high purity and cytolytic activity *in vitro* [13].

The present study was designed to pre-clinically evaluate the *in vivo* anti-leukemic potential of UCB-NK cells generated with our GMP-compliant culture system. To this end, we developed a mouse model in which human K562.LucGFP leukemia cells are directly implanted into the femur of NSG mice, thus requiring BM-specific homing of infused UCB-NK cells to achieve anti-tumor response. In this model, we showed that treatment with UCB-NK cells in combination with supportive IL-15 potently inhibited progression of K562 cells, thereby demonstrating that UCB-NK cells are functional *in vivo* and have the capacity to target leukemia cells within BM upon adoptive transfer. Importantly, prolongation of mice survival including complete and persistent response in 25% of the mice was achieved following a single infusion of UCB-NK cells and in the presence of low dose IL-15 support. Considering that complete protection against K562 cells *in vivo* was mostly reported following multiple NK cell infusions and/or prolonged high-dose IL-2 administration [21]–[24], we believe that our results nicely illustrates the therapeutic potential of UCB-NK cells in the clinic. In addition, biodistribution analysis showed that a relatively small proportion of our current UCB-NK cell product accumulates in one femur within 24 h (∼0.3% of 20×10^6^ cells, i.e. <1×10^5^ UCB-NK cells per femur), suggesting that inhibition of leukemia cell growth primarily happened at a low effector to target ratio what also illustrates the high cytolytic potential of *ex vivo* generated UCB-NK cells.

Our phenotypical analysis and *in vitro* migration studies support that UCB-NK cells can actively home to BM following adoptive transfer. Consistent expression of the chemokine receptor CXCR4 was detected on UCB-NK cells at the end of the culture process, and robust migration in response to its ligand CXCL12 was shown *in vitro*. Implication of the CXCR4/CXCL12 axis in regulating the migration of human T and NK cells to BM has been reported in several studies [25], [26], including in human metastatic prostate cancer [27]. Therefore, it is likely that UCB-NK cell homing to BM is CXCR4/CXCL12-dependent. Nevertheless, we also showed that UCB-NK cells display high expression of CXCR3 and CCR6, two receptors that could also regulate UCB-NK cell trafficking to inflamed tissues *in vivo*. Future studies are now warranted to demonstrate the role of the CXCR4/CXCL12 axis in UCB-NK cell homing to BM and to examine its implication in patients among other inflammatory pathways like CXCR3/CXCL10–11. Also, trafficking of adoptively transferred NK cells has not been addressed in leukemia patients yet. Use of ^111^In for *in vivo* tracking of adoptively transferred NK cells has already been reported in patients with renal cell carcinoma and liver metastases [28], [29], and at the pre-clinical level in xenograft mouse models of leukemia [23]. Similar to our findings, early distribution of NK cells in the lungs, and later in the liver, spleen and eventually BM was visualized following systemic infusion. However, detailed organ distribution of NK cells as well as demonstration of the specificity of the visualized signal, particularly in BM, were not available. Here, we showed that ^111^In-based NK cell tracking provides good specificity and sensitivity, and will constitute a useful method to study and correlate effective BM targeting with clinical response in patients.

We reported previously that UCB-NK cells generated with our GMP-compliant culture system display a relatively low expression level of CD16 and KIRs at the end of the culture process [12]. However, we observed that the proportions of UCB-NK cells expressing these markers were strongly increased two weeks after adoptive transfer, indicating that UCB-NK cells can further differentiate *in vivo* into a more mature cell population. These data are in agreement with Huntington et al. who showed that rapid IL-15 driven-transition of human CD56^hi^CD16^−^KIR^−^ to CD56^dim^CD16^+^KIR^+^ NK cells occurs *in vivo* [30]. Since KIR-ligand mismatch triggers NK cell alloreactivity towards AML, such phenotypic modifications might have important implications in patients. In that view, UCB-NK cell reactivity towards AML *in vivo* should be further addressed using HLA-expressing AML cells in our *i.f.* NSG model.

In conclusion, our results strongly support that UCB-NK cells constitute promising immunotherapeutic products to improve the treatment of AML, as demonstrated by their capability to migrate to BM and to inhibit progression of human leukemia cells following adoptive transfer. In addition, we demonstrated efficient UCB-NK survival *in vivo* in the presence of low dose human IL-15. Transient elevation of IL-15 plasma levels was reported in AML patients following immunosuppressive Cy/Flu conditioning [9], [10], what could favor *in vivo* expansion and maturation of UCB-NK cells as well as clinical responses following UCB-NK cell adoptive transfer. Since BM is the primary site of AML development and encloses niches essential for leukemic stem cells causing relapse [31], we believe that BM targeting is essential for elimination of minimal residual disease and induction of optimal and persistent clinical responses against AML. Strategies that aim at increasing BM-specific chemokine receptors, like CXCR4, on UCB-NK cells are now considered to enhance BM targeting. In addition, the methodologies that we report here for UCB-NK cell tracking and anti-leukemic effect monitoring will be instrumental to validate future findings and to fully exploit the potential of UCB-NK cells against AML and other hematological malignancies.

## Supporting Information

Figure S1 **The biodistribution of ^111^In-NK cells upon adoptive transfer is reproducible between animals and independent of the dose of injected cells.** Five adult NSG mice (mice M1 to M5) were infused *i.v.* with increasing number of ^111^In-NK cells (1MBq per 10^6^ cells) and euthanized the day after for organ collection and biodistribution analysis. For comparison, proportions of activity quantified per gram of tissue of interest were normalized to blood.
(TIF)

Figure S2 **Low-dose IL-15 mediates efficient UCB-NK cell survival and expansion** ***in vivo*** **.** Adult NSG mice were injected *i.v.* with 10×10^6^ UCB-NK cells with or without supportive IL-15. Recombinant human IL-15 was administered daily for 2 weeks at the dose of 0.5 µg/mouse/injection, starting the day of UCB-NK cell infusion. Human NK cells were quantified weekly in peripheral blood by flow cytometric analysis. (A) Percentage of human CD45^+^CD56^+^ cells in blood of mice injected with UCB-NK cells alone (dotted line, n = 5) or UCB-NK cells with IL-15 (straight line, n = 6) over time. (B) Representative dot-plots obtained 2 weeks after UCB-NK cell infusion.
(TIF)

## References

[1] Rowe JM (2009) Closer to the truth in AML. Blood 113: 4129–4130. 19406994 10.1182/blood-2008-12-192856

[2] Estey E, Dohner H (2006) Acute myeloid leukaemia. Lancet 368: 1894–1907. 17126723 10.1016/S0140-6736(06)69780-8

[3] Jemal A, Siegel R, Xu J, Ward E (2010) Cancer statistics, 2010. CA Cancer J Clin 60: 277–300. 20610543 10.3322/caac.20073

[4] Buchner T, Berdel WE, Wormann B, Schoch C, Haferlach T, et al (2005) Treatment of older patients with AML. Crit Rev Oncol Hematol 56: 247–259. 16246568 10.1016/j.critrevonc.2004.09.010

[5] Lowenberg B, Downing JR, Burnett A (1999) Acute myeloid leukemia. N Engl J Med 341: 1051–1062. 10502596 10.1056/NEJM199909303411407

[6] Vivier E, Tomasello E, Baratin M, Walzer T, Ugolini S (2008) Functions of natural killer cells. Nat Immunol 9: 503–510. 18425107 10.1038/ni1582

[7] Caligiuri MA (2008) Human natural killer cells. Blood 112: 461–469. 18650461 10.1182/blood-2007-09-077438 PMC2481557

[8] Ruggeri L, Mancusi A, Capanni M, Urbani E, Carotti A, et al (2007) Donor natural killer cell allorecognition of missing self in haploidentical hematopoietic transplantation for acute myeloid leukemia: challenging its predictive value. Blood 110: 433–440. 17371948 10.1182/blood-2006-07-038687 PMC1896125

[9] Miller JS, Soignier Y, Panoskaltsis-Mortari A, McNearney SA, Yun GH, et al (2005) Successful adoptive transfer and in vivo expansion of human haploidentical NK cells in patients with cancer. Blood 105: 3051–3057. 15632206 10.1182/blood-2004-07-2974

[10] Curti A, Ruggeri L, D'Addio A, Bontadini A, Dan E, et al (2011) Successful transfer of alloreactive haploidentical KIR ligand-mismatched natural killer cells after infusion in elderly high risk acute myeloid leukemia patients. Blood 118: 3273–3279. 21791425 10.1182/blood-2011-01-329508

[11] Rubnitz JE, Inaba H, Ribeiro RC, Pounds S, Rooney B, et al (2010) NKAML: a pilot study to determine the safety and feasibility of haploidentical natural killer cell transplantation in childhood acute myeloid leukemia. J Clin Oncol 28: 955–959. 20085940 10.1200/JCO.2009.24.4590 PMC2834435

[12] Spanholtz J, Tordoir M, Eissens D, Preijers F, van der Meer A, et al (2010) High log-scale expansion of functional human natural killer cells from umbilical cord blood CD34-positive cells for adoptive cancer immunotherapy. PLoS One 5: e9221. 20169160 10.1371/journal.pone.0009221 PMC2821405

[13] Spanholtz J, Preijers F, Tordoir M, Trilsbeek C, Paardekooper J, et al (2011) Clinical-grade generation of active NK cells from cord blood hematopoietic progenitor cells for immunotherapy using a closed-system culture process. PLoS One 6: e20740. 21698239 10.1371/journal.pone.0020740 PMC3116834

[14] Boggs DR (1984) The total marrow mass of the mouse: a simplified method of measurement. Am J Hematol 16: 277–286. 6711557 10.1002/ajh.2830160309

[15] Lehmann D, Spanholtz J, Osl M, Tordoir M, Lipnik K, et al (2012) Ex vivo generated natural killer cells acquire typical natural killer receptors and display a cytotoxic gene expression profile similar to peripheral blood natural killer cells. Stem Cells Dev 21: 2926–2938. 22571679 10.1089/scd.2011.0659 PMC3475144

[16] Campbell JJ, Qin S, Unutmaz D, Soler D, Murphy KE, et al (2001) Unique subpopulations of CD56+ NK and NK-T peripheral blood lymphocytes identified by chemokine receptor expression repertoire. J Immunol 166: 6477–6482. 11359797 10.4049/jimmunol.166.11.6477

[17] Berahovich RD, Lai NL, Wei Z, Lanier LL, Schall TJ (2006) Evidence for NK cell subsets based on chemokine receptor expression. J Immunol 177: 7833–7840. 17114454 10.4049/jimmunol.177.11.7833

[18] Nguyen S, Beziat V, Norol F, Uzunov M, Trebeden-Negre H, et al (2011) Infusion of allogeneic natural killer cells in a patient with acute myeloid leukemia in relapse after haploidentical hematopoietic stem cell transplantation. Transfusion 51: 1769–1778. 21332732 10.1111/j.1537-2995.2010.03058.x

[19] Tanaka J, Sugita J, Shiratori S, Shigematu A, Asanuma S, et al (2012) Expansion of NK cells from cord blood with antileukemic activity using GMP-compliant substances without feeder cells. Leukemia 26: 1149–1152. 22143670 10.1038/leu.2011.345

[20] Yoon SR, Lee YS, Yang SH, Ahn KH, Lee JH, et al (2010) Generation of donor natural killer cells from CD34(+) progenitor cells and subsequent infusion after HLA-mismatched allogeneic hematopoietic cell transplantation: a feasibility study. Bone Marrow Transplant 45: 1038–1046. 19881555 10.1038/bmt.2009.304

[21] Woll PS, Grzywacz B, Tian X, Marcus RK, Knorr DA, et al (2009) Human embryonic stem cells differentiate into a homogeneous population of natural killer cells with potent in vivo antitumor activity. Blood 113: 6094–6101. 19365083 10.1182/blood-2008-06-165225 PMC2699231

[22] Ayello J, van de Ven C, Fortino W, Wade-Harris C, Satwani P, et al (2006) Characterization of cord blood natural killer and lymphokine activated killer lymphocytes following ex vivo cellular engineering. Biol Blood Marrow Transplant 12: 608–622. 16737934 10.1016/j.bbmt.2006.01.009

[23] Guimaraes F, Guven H, Donati D, Christensson B, Ljunggren HG, et al (2006) Evaluation of ex vivo expanded human NK cells on antileukemia activity in SCID-beige mice. Leukemia 20: 833–839. 16511516 10.1038/sj.leu.2404147

[24] Fujisaki H, Kakuda H, Shimasaki N, Imai C, Ma J, et al (2009) Expansion of highly cytotoxic human natural killer cells for cancer cell therapy. Cancer Res 69: 4010–4017. 19383914 10.1158/0008-5472.CAN-08-3712 PMC2716664

[25] Beider K, Nagler A, Wald O, Franitza S, Dagan-Berger M, et al (2003) Involvement of CXCR4 and IL-2 in the homing and retention of human NK and NK T cells to the bone marrow and spleen of NOD/SCID mice. Blood 102: 1951–1958. 12730102 10.1182/blood-2002-10-3293

[26] Pinthus JH, Waks T, Malina V, Kaufman-Francis K, Harmelin A, et al (2004) Adoptive immunotherapy of prostate cancer bone lesions using redirected effector lymphocytes. J Clin Invest 114: 1774–1781. 15599402 10.1172/JCI22284 PMC535069

[27] Zhao E, Wang L, Dai J, Kryczek I, Wei S, et al (2012) Regulatory T cells in the bone marrow microenvironment in patients with prostate cancer. Oncoimmunology 1: 152–161. 22720236 10.4161/onci.1.2.18480 PMC3376984

[28] Brand JM, Meller B, Von Hof K, Luhm J, Bahre M, et al (2004) Kinetics and organ distribution of allogeneic natural killer lymphocytes transfused into patients suffering from renal cell carcinoma. Stem Cells Dev 13: 307–314. 15186726 10.1089/154732804323099235

[29] Matera L, Galetto A, Bello M, Baiocco C, Chiappino I, et al (2006) In vivo migration of labeled autologous natural killer cells to liver metastases in patients with colon carcinoma. J Transl Med 4: 49. 17105663 10.1186/1479-5876-4-49 PMC1681349

[30] Huntington ND, Legrand N, Alves NL, Jaron B, Weijer K, et al (2009) IL-15 trans-presentation promotes human NK cell development and differentiation in vivo. J Exp Med 206: 25–34. 19103877 10.1084/jem.20082013 PMC2626663

[31] Ishikawa F, Yoshida S, Saito Y, Hijikata A, Kitamura H, et al (2007) Chemotherapy-resistant human AML stem cells home to and engraft within the bone-marrow endosteal region. Nat Biotechnol 25: 1315–1321. 17952057 10.1038/nbt1350

