# Supplementary figures and images for: Natural Killer Cells Generated from Cord Blood Hematopoietic Progenitor Cells Efficiently Target Bone Marrow-Residing Human Leukemia Cells in NOD/SCID/IL2Rgnull Mice
Source: PLoS One. 2013 Jun 5;8(6):e64384. doi: 10.1371/journal.pone.0064384 (PMC3673996; doi:10.1371/journal.pone.0064384)

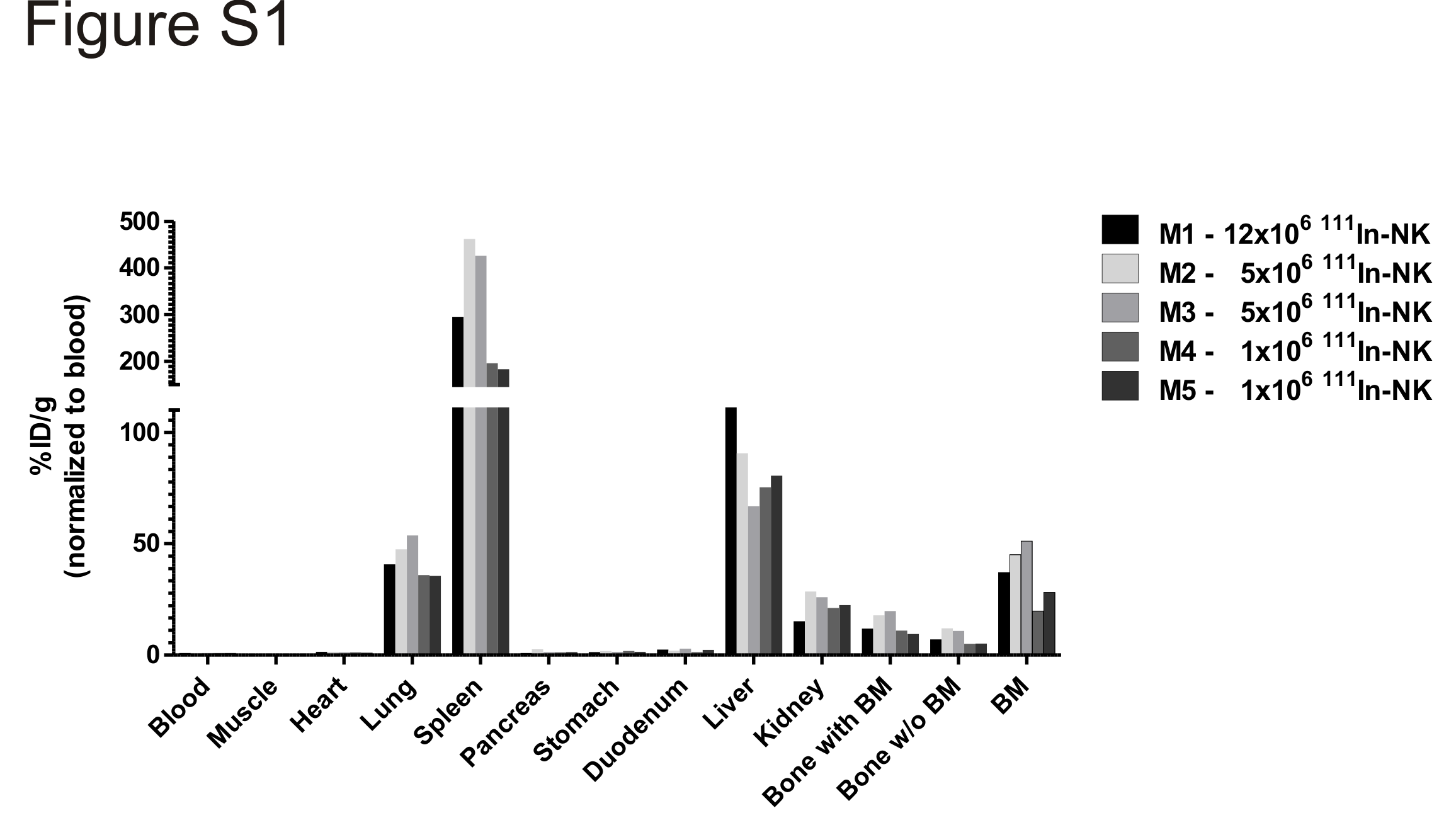

Supplement: Figure S1 — The biodistribution of 111In-NK cells upon adoptive transfer is reproducible between animals and independent of the dose of injected cells. Five adult NSG mice (mice M1 to M5) were infused i.v. with increasing number of 111In-NK cells (1MBq per 106 cells) and euthanized the day after for organ collection and biodistribution analysis. For comparison, proportions of activity quantified per gram of tissue of interest were normalized to blood. (TIF) [file pone.0064384.s001.tif]

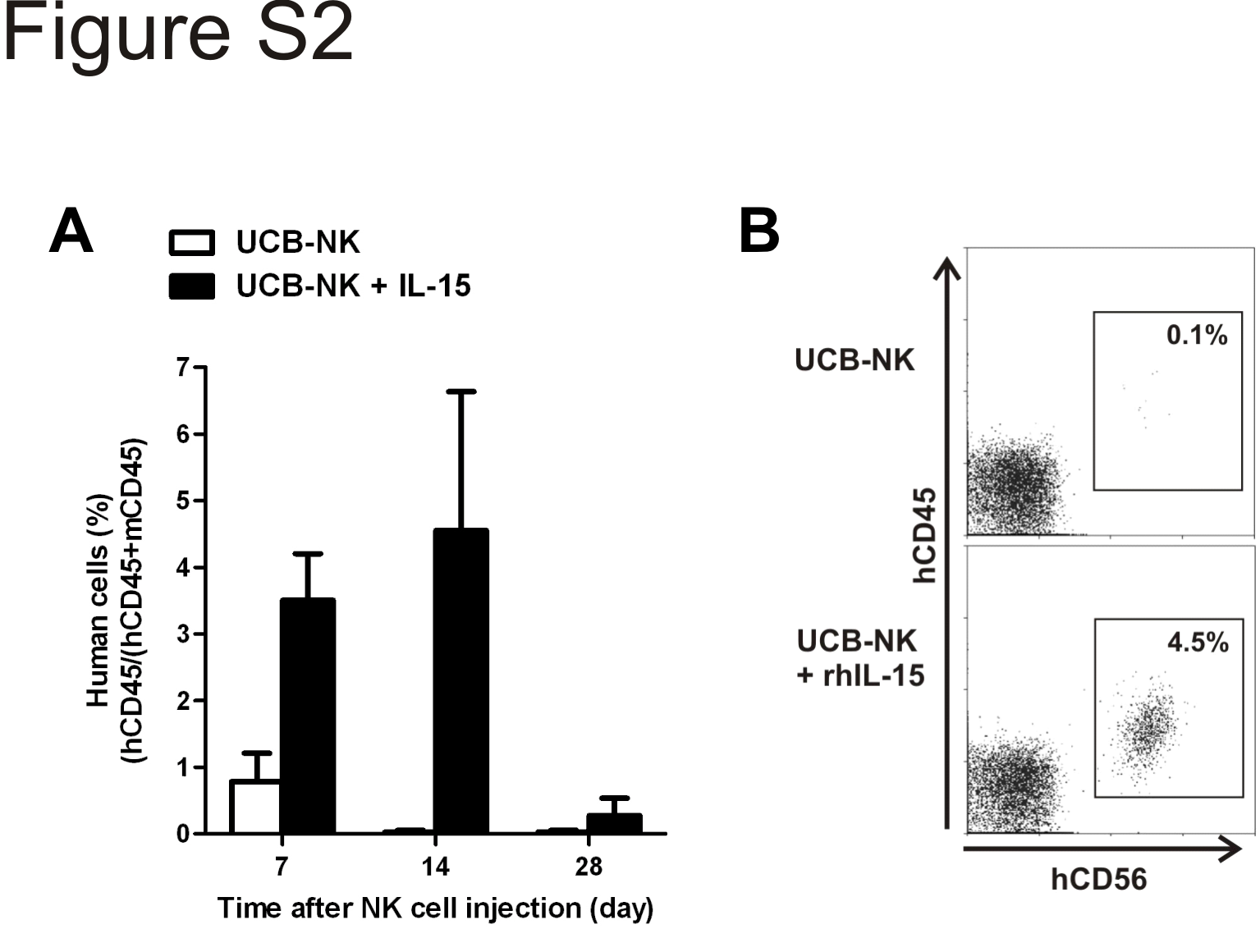

Supplement: Figure S2 — Low-dose IL-15 mediates efficient UCB-NK cell survival and expansion in vivo . Adult NSG mice were injected i.v. with 10×106 UCB-NK cells with or without supportive IL-15. Recombinant human IL-15 was administered daily for 2 weeks at the dose of 0.5 µg/mouse/injection, starting the day of UCB-NK cell infusion. Human NK cells were quantified weekly in peripheral blood by flow cytometric analysis. (A) Percentage of human CD45+CD56+ cells in blood of mice injected with UCB-NK cells alone (dotted line, n = 5) or UCB-NK cells with IL-15 (straight line, n = 6) over time. (B) Representative dot-plots obtained 2 weeks after UCB-NK cell infusion. (TIF) [file pone.0064384.s002.tif]
